# The relationship between high-dose corticosteroid treatment and mortality in acute respiratory distress syndrome: a retrospective and observational study using a nationwide administrative database in Japan

**DOI:** 10.1186/s12890-018-0597-5

**Published:** 2018-02-07

**Authors:** Takashi Kido, Keiji Muramatsu, Takeshi Asakawa, Hiroki Otsubo, Takaaki Ogoshi, Keishi Oda, Tatsuhiko Kubo, Yoshihisa Fujino, Shinya Matsuda, Toshihiko Mayumi, Hiroshi Mukae, Kazuhiro Yatera

**Affiliations:** 1 Department of Respiratory Medicine, University of Occupational and Environmental Health, 1-1 Iseigaoka, Yahatanishi-ku, Kitakyushu, Japan; 2 Department of Emergency Medicine, University of Occupational and Environmental Health, Kitakyushu, Japan; 3 Department of Preventive Medicine and Community Health, University of Occupational and Environmental Health, Kitakyushu, Japan; 4 Department of Information Systems Center, University of Occupational and Environmental Health, Kitakyushu, Japan; 5 Second Department of Internal Medicine, Nagasaki University Hospital, Nagasaki, Japan

**Keywords:** Acute respiratory distress syndrome, Corticosteroid, Inverse probability of treatment weighting method, Nationwide administrative database, Propensity score

## Abstract

**Background:**

In the 1980s, randomized-controlled trials showed that high-dose corticosteroid treatment did not improve the mortality of acute respiratory distress syndrome (ARDS). However, while the diagnostic criteria for ARDS have since changed, and supportive therapies have been improved, no randomized-controlled trials have revisited this issue since 1987; thus, the effect of high-dose corticosteroid treatment may be different in this era. We evaluated the effect of high-dose corticosteroid treatment in patients with ARDS using a nationwide administrative database in Japan in a retrospective and observational study.

**Methods:**

This study was performed with a large population using the 2012 Japanese nationwide administrative database (diagnostic procedure combination). We evaluated the mortality of ARDS patients receiving or not receiving high-dose corticosteroid treatment within 7 days of hospital admission. We employed propensity score weighting with a Cox proportional hazards model in order to minimize the bias associated with the retrospective collection of data on baseline characteristics and compared the mortality between the high-dose and non-high-dose corticosteroid groups.

**Results:**

Data from 2707 patients were used; 927 patients were treated with high-dose corticosteroid and 1780 patients were treated without high-dose corticosteroid, within 7 days of admission. After adjusting for confounds, mortality rates within 3 months were significantly higher in the high-dose corticosteroid group compared to the non-high-dose corticosteroid group (weighted hazard ratio: 1.59; 95% CI: 1.37-1.84; *P* <  0.001).

**Conclusions:**

Our results suggest that high-dose corticosteroid treatment does not improve the prognosis of patients with ARDS, even in this era. However, this study has limitations owing to its retrospective and observational design.

## Background

Acute respiratory distress syndrome (ARDS) is a critical respiratory syndrome. Recent advances in treatment strategies, such as protective mechanical ventilation techniques, have improved the mortality of patients with ARDS, according to many clinical trials [1–4]. However, no pharmacotherapies have yet been shown to be effective in improving the mortality rate; according to systematic reviews, mortalities due to ARDS were as high as 43 and 44% in 2008 and 2009, respectively, [2, 5].

A systemic inflammatory response is closely associated with the development of ARDS. Thus, anti-inflammatory corticosteroid treatment may be a logical choice for ARDS [6–8]. However, according to previous studies and meta-analyses, the efficacy of corticosteroids in ARDS is still controversial; while some reports have shown improvement in the mortality [9–13], others have not [14–16]. Furthermore, although meta-analyses have indicated the poor effectiveness of corticosteroids, they also highlight the difficulties in confirming the role of corticosteroids due to the heterogeneity of ARDS studies [8, 17]. The dosage of corticosteroids for the treatment of ARDS is also controversial. In the 1980s, a few randomized-controlled trials (RCTs) showed that high-dose corticosteroids did not improve mortality, and subsequently, for almost three decades, no RCTs have revisited this topic due to the results of these reports [14, 18]. However, the ARDS diagnostic criteria, supportive methods, and treatments for underlying diseases have changed over the years. In 1994, the American-European Consensus Conference (AECC) resulted in the development of new diagnostic criteria for ARDS [19], which were subsequently modified (Berlin definition [20]). We therefore speculated that the effectiveness of high-dose corticosteroid treatment might be different in the present era. In this retrospective and observational study, we investigated the effectiveness of high-dose corticosteroid treatment using the Japanese nationwide administrative database: the diagnostic procedure combination (DPC). To minimize the bias associated with the retrospective collection of data (i.e. the baseline characteristics in the high-dose and non-high-dose corticosteroid groups), we employed propensity score weighting with a Cox proportional hazards model [21–25].

## Methods

### Data source

The DPC is a case-mix patient classification system that was introduced by the Japanese government in 2002 and is linked with a lump-sum payment system [26]. It covers approximately 40% of all acute-care hospitalizations in Japan and has been actively utilized for the evaluation of treatments [27–29]. The database contains the following information: disease name, treatment costs, comorbid illnesses at admission and during hospitalization (coded by the *International Classification of Diseases, 10th revision*; ICD-10), patients’ age, sex, length of stay, medical procedures, intensive-care unit (ICU) admission, interventional procedures (including mechanical ventilation and hemodialysis), medications, state of consciousness according to the Japan Coma Scale (JCS) on admission, and discharge status (including in-hospital deaths) [26, 29].

Any patient identifiable information was removed from the data. This study was conducted according to guidelines laid down in the Declaration of Helsinki and was approved by Ethics Committee of Medical Research, University of Occupational and Environmental Health, Japan. Informed consent was waived because of the retrospective study design.

### Patient selection

Patients who were diagnosed with ARDS, ICD-10 code J80 or pneumonia at admission (and subsequently diagnosed with ARDS as the predominant reason for hospitalization as indicated by the cost during hospitalization) and discharged within 2012 were included. Patients who were discharged and died within 7 days of hospitalization or who did not receive mechanical ventilation were excluded, as we believed that if the duration of administration was too short, the effect of high-dose corticosteroid on ARDS could not be analyzed properly, and the use of mechanical ventilation is necessary for the diagnosis according to the criteria of ARDS [19, 20].

### Variables

Patients’ sex and age (years), hospital volume (number of patients with ARDS treated in 2012), emergency transport, diagnoses of sepsis, cancer, pneumonia, pancreatitis, lung or abdominal trauma, liver dysfunction (diagnosed as liver failure, hepatitis, or liver cirrhosis) at admission, hemodialysis performed within 7 days of admission, neurological dysfunction (JCS at admission of ≥100 indicating coma) [29], shock (use of a vasopressor within 7 days of admission), medication use (insulin, antithrombin III, recombinant human soluble thrombomodulin, heparin, synthetic protease inhibitors, or sivelestat within 7 days from admission), transfusion of platelets and red cells within 7 days of admission, administration of albumin and immunoglobulin within 7 days of admission, mechanical ventilation, and ICU transfer within 7 days of admission were used as variables.

### Statistical analyses

The high-dose corticosteroid group was defined as the patients who received treatment with methylprednisolone at doses of > 500 mg/day for > 1 day within 7 days of admission. The definition of high-dose corticosteroid was taken from previous meta-analyses [8], and infusion of 500 mg of methylprednisolone every 12 h for < 72 h is the most common regimen of high-dose corticosteroid therapy in Japan.

The primary outcome was mortality; the secondary endpoints were the duration (in days) for which mechanical ventilation was used and the duration of ICU stay within the 28 days after admission. We employed propensity score weighting with a Cox proportional hazards model, as described previously [8, 25, 29, 30]. The propensity score was calculated using a logistic model with baseline variables that potentially influenced the use of high-dose corticosteroid, including patients’ sex and age, hospital volume, sepsis, cancer, pneumonia, pancreatitis, lung and abdominal trauma, liver dysfunction, hemodialysis, neurological dysfunction, shock, use of antithrombin III, recombinant human soluble thrombomodulin, heparin, synthetic protease inhibitors and sivelestat, platelet and red cell transfusions, albumin and immunoglobulin administration, and mechanical ventilation status.

The C-statistic was used to evaluate goodness of fit. To check the balance of the measured covariates, χ^2^ or Fisher’s exact tests were used for categorical data, and unpaired *t*-tests or Mann-Whitney U tests were used for continuous variables to evaluate the between-group (high-dose vs. non-high-dose corticosteroid group) differences before and after adjusting for confounders using propensity score weighting. The adjusted Kaplan-Meier curves were depicted, and the adjusted hazard ratio (HR) and robust 95% confidence interval (CI) were estimated in a Cox regression model [8, 25, 30, 31]. This method was performed using the IBM SPSS 22.0 (Armonk, NY, USA) and STATA/IC 14.0 (StataCorp, College Station, TX, USA) software programs. Differences of *P* <  0.05 were considered statistically significant in all tests.

## Results

**Fig. 1:**
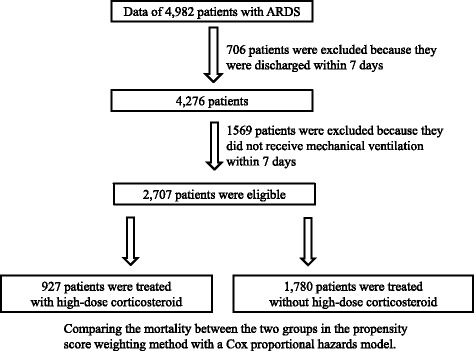
Flowchart of this study. Among the 4982 patients diagnosed with ARDS in the 2012 Japanese nationwide administrative database (diagnostic procedure combination), the data of the 2707 patients who met the inclusion criteria were used. Of these 2707 patients, 927 received high-dose corticosteroid treatment, and 1780 received non-high-dose corticosteroid treatment within 7 days of admission. We employed propensity score weighting with a Cox proportional hazards model in order to minimize the bias associated with the retrospective collection of data on baseline characteristics and compared the mortality between the two groups

Among the data of 4982 patients diagnosed with ARDS in the DPC database (as described in the Methods), the data of 706 patients were excluded because the patients were discharged within 7 days of admission. The data of 1569 patients were also excluded because the patients did not receive mechanical ventilation within 7 days of admission. Of the remaining 2707 patients, 927 received high-dose corticosteroid treatment, and 1780 received non-high-dose corticosteroid treatment within 7 days of admission (Fig. 1).

**Fig. 2:**
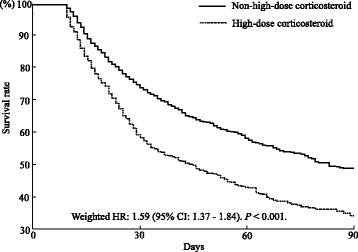
Adjusted Kaplan-Meier survival curves. In the propensity score-weighted Cox proportional hazards model, the mortality rate of the high-dose corticosteroid group was significantly lower than that of the non-high-dose corticosteroid group (weighted HR: 1.59; 95% CI: 1.37-1.84; *P* <  0.001)

To minimize the bias associated with the retrospective collection of data in the high-dose and non-high-dose corticosteroid groups, we employed propensity score weighting with a Cox proportional hazards model. The C-statistic (area under the receiver operating characteristic curve) of the propensity score was 0.72. Patient baseline characteristics, before and after adjusting for confounders, are shown in Table 1. Before adjustment, the baseline variables of patient age, hospital volume, emergency transport, sepsis, cancer, pneumonia, pancreatitis, lung and abdominal trauma, hemodialysis, neurological dysfunction, shock, insulin, antithrombin III, recombinant human soluble thrombomodulin, platelet and red cell transfusions, albumin administration, immunoglobulin administration, and ICU transfer were significantly different between the high-dose and non-high-dose corticosteroid groups. After adjusting for confounders using the IPTW method, patient baseline characteristics between the two groups were similar across these variables, although pneumonia, pancreatitis, lung and abdominal trauma, neurological dysfunction, and insulin were still significantly different between the groups. In the propensity score-weighted Cox proportional hazards model, the mortality was significantly lower in the high-dose corticosteroid group than in the non-high-dose corticosteroid group (weighted HR: 1.59; 95% CI: 1.37-1.84; *P* <  0.001; Fig. 2). There were no significant differences between the two groups with regard to the duration for which mechanical ventilation was used (3.5 ± 0.2 versus 3.3 ± 0.1 days, *P* = 0.323), and the duration of the ICU stay (13.2 ± 0.3 versus 12.8 ± 0.2 days, *P* = 0.330) within the 28 days after admission (Table 2).

**Table 1:** Baseline characteristics of the patients treated with or without high-dose corticosteroid before and after group adjustment

	Before adjustment			After adjustment		
	High-dose corticosteroid (*n* = 927)	Non-high-dose corticosteroid (*n* = 1780)	*p-value*	High-dose corticosteroid (*n* = 927)	Non-high-dose corticosteroid (*n* = 1780)	*p-value*
Sex	68.1	66.9	0.562	68.1	67.8	0.771
Age (years)	71.6 ± 0.5	67.9 ± 0.5	< 0.001	67.7 ± 1.0	69.3 ± 0.4	0.05
Hospital volume per year	10.3 ± 0.3	13.0 ± 0.3	< 0.001	12.2 ± 0.6	12.1 ± 0.2	0.806
Sepsis	12.1	22.1	< 0.001	19.9	19	0.373
Cancer	12.3	8.3	0.001	10.2	9.7	0.487
Pneumonia	52.6	62.4	< 0.001	63.6	60.3	< 0.001
Pancreatitis	0.3	1.1	0.041	0.4	0.8	0.001
Lung and abdominal trauma	0.1	0.7	0.043	0.2	0.4	0.009
Liver dysfunction	2.1	2.2	0.88	3.0	2.2	0.392
Hemodialysis	10.1	13.1	0.023	14.0	12.4	0.085
Neurological dysfunction	10.6	22.5	< 0.001	21.8	18.8	0.034
Shock	44.2	48.1	0.053	47.4	47.2	0.848
Insulin	61.7	45.9	< 0.001	48.7	51.2	0.016
Antithrombin III	9.0	15.4	< 0.001	13.9	13.2	0.537
rhTM	10.0	14.1	0.002	14.6	12.9	0.159
Heparin	58.2	61.5	0.094	62.8	61.7	0.088
Protease inhibitors	17.8	19.4	0.318	20.1	19.2	0.331
Sivelestat	72.9	56.2	< 0.001	59.7	61.8	0.074
Platelet transfusion	8.0	10.2	0.045	9.7	9.7	0.938
Red blood cell transfusion	16.4	26.5	< 0.001	24.8	23.3	0.241
Albumin administration	31.7	41.6	< 0.001	38.9	38.5	0.706
Immunoglobulin administration	18.1	23.5	0.001	22.2	21.8	0.637
Intensive-care unit	33.3	32.9	0.852	33.7	33.4	0.754

Data are presented as the % or mean ± standard error, unless otherwise stated. Groups were adjusted using the inverse probability of treatment weighting method

*rhTM* recombinant human soluble thrombomodulin

**Table 2:** Secondary endpoints of the patients treated with or without high-dose corticosteroids before and after group adjustment.

	Before adjustment			After adjustment		
	High-dose corticosteroid (*n* = 927)	Non-high-dose corticosteroid (*n* = 1780)	*p-value*	High-dose corticosteroid (*n* = 927)	Non-high-dose corticosteroid (*n* = 1780)	*p-value*
Duration of mechanical ventilation use within 28 days (days)	13.3 ± 0.2	12.9 ± 0.2	0.108	13.2 ± 0.3	12.8 ± 0.2	0.330
Duration of intensive-care unit stay within 28 days (days)	3.4 ± 0.2	3.3 ± 0.1	0.863	3.5 ± 0.2	3.3 ± 0.1	0.323

Data are presented as the mean ± standard error. Groups were adjusted using the inverse probability of treatment weighting method

## Discussion

To date, no pharmacotherapies have demonstrated robust, beneficial effects on the outcomes of patients with ARDS. In the present study, we observed the effects of high-dose corticosteroid treatment in a large number of Japanese patients with ARDS using a Japanese nationwide administrative database (DPC) by propensity score weighting with a Cox proportional hazards model. After adjusting for baseline characteristics, the mortality was found to be significantly worse in patients who received high-dose corticosteroid treatment than in those who received non-high-dose corticosteroid treatment.

The efficacy of corticosteroids is still controversial; some reports have shown improvements in mortality [9–13], while others have not [14–16]. Our results showed a higher mortality following high-dose corticosteroid use than without the use of such agents. Previous reports of the high-dose corticosteroid treatment showed that high-dose corticosteroid did not improve the mortality [7, 8, 13, 14, 16, 17, 32]. Among these, Bernard et al. showed that 30 mg/kg of body weight of methylprednisolone every 6 h for 24 h did not improve the mortality of patients with ARDS [14]. Very recently, a meta-analysis showed that high-dose corticosteroid treatment did not improve the mortality [13], and a recent retrospective propensity-matched study also showed that high-dose corticosteroid treatment increased the mortality compared to low-dose corticosteroid treatment [32]. However, the reasons for this are unclear. Weigelt et al. showed that high-dose corticosteroid treatment (30 mg/kg of body weight every 6 h for 48 h) did not prevent patients with respiratory failure from developing ARDS but did increase the risk of infectious complications [33]. In our patients, approximately 60 and 20% of patients were diagnosed with pneumonia and sepsis, respectively, at the time of admission. Thus, high-dose corticosteroids may have exacerbated infectious diseases or increased infectious complications in the present study, similar to a recent study by Takaki et al. [32]. Other than mortality, we have also shown the effects of corticosteroids on the duration for which mechanical ventilation is used and the duration of the ICU stay within the 28 days after admission, which did not differ between the groups to a statistically significant extent. On the other hand, several studies that have investigated lower dosages of corticosteroids and different protocols have shown the effects on shortening the duration of mechanical ventilation usage and ICU stay [12, 15]. Further studies might be needed to investigate the effects of high-dose corticosteroids on these secondary outcomes.

Propensity score weighting has recently been used in observational studies to assess the effect of treatment after adjusting for baseline characteristics in order to minimize the drawbacks associated with the propensity score matching method, such as sampling biases and loss of sample numbers. ARDS is relatively rare and clinically severe; therefore, it may be not easy to perform new and large RCTs, especially for the reevaluation of clinically used medications that have the potential to be clinically effective in different scenarios from previous studies. Even in retrospective studies, we speculate that propensity score weighting with a Cox proportional hazards model and a large subject population is suitable for evaluating the clinical effect of certain medications, such as high-dose corticosteroid, in patients with ARDS.

There are several limitations associated with this study, similar to previous studies using the DPC database [27–29, 34]. First, this study was observational and retrospective; however, our application of propensity score weighting with a Cox proportional hazards model reduced the effects of this limitation. Second, even after adjustment, there were still significant differences in the characteristics of the high-dose and non-high-dose corticosteroid groups, such as in the rates of pneumonia, pancreatitis, lung and abdominal trauma, neurological dysfunction and insulin. However, after adjusting for confounders, the differences between the two groups were small. For example, the frequency of neurological dysfunction in the high-dose corticosteroid and non-high-dose corticosteroid groups were 10.6% versus 22.5%, respectively, before adjustment, and 21.8% versus 18.8% after adjustment. While this remains one of the limitations of the present study, we consider these differences to be relatively small. Third, we were unable to include several clinical data, such as the peripheral blood laboratory findings, radiological findings, physiological data, including vital signs, and mechanical ventilation settings, which could also influence the mortality in patients with ARDS. Fourth, the ARDS diagnostic criteria have changed over the years [19, 20], so it was unclear which criteria had been used to diagnose ARDS in each patient in this retrospective study.

Despite these limitations, the major advantages of this study were the inclusion of a large number of patients and evaluation of the effect of high-dose corticosteroid treatment in this era. To our knowledge, this is the largest study of high-dose corticosteroid treatment for ARDS, even compared to previous meta-analyses [7, 8, 13, 17].

## Conclusions

We observed a higher mortality with the administration of high-dose corticosteroids within 7 days of hospital admission in patients with ARDS than without the administration of such agents. We used a nationwide administrative database, making this the largest study to observe high-dose corticosteroid treatment for ARDS, to our knowledge. However, this study has some limitations owing to its retrospective and observational design.

## Abbreviations

AECC: American-European Consensus Conference
ARDS: Acute respiratory distress syndrome
CI: Confidence interval
DPC: Diagnostic procedure combination
HR: Hazard ratio
ICD-10: International Classification of Diseases, 10th revision
ICU: Intensive-care unit
IPTW: Inverse probability of treatment weighting
JCS: Japan Coma Scale
RCT: Randomized controlled trial
